# The neutrophil-to-lymphocyte ratio may indicate when to start hemodialysis

**DOI:** 10.1080/0886022X.2022.2110894

**Published:** 2022-08-15

**Authors:** Tae Won Lee, Wooram Bae, Jungyoon Choi, Eunjin Bae, Ha Nee Jang, Se-Ho Chang, Dong Jun Park

**Affiliations:** a Department of Internal Medicine, Gyeongsang National University Changwon Hospital, Changwon, South Korea; b Department of Internal Medicine, Gyeongsang National University College of Medicine, Jinju, South Korea; c Institute of Health Science, Gyeongsang National University, Jinju, South Korea; d Department of Internal Medicine, Gyeongsang National University Hospital, Jinju, South Korea

**Keywords:** Hemodialysis, uremia, end stage kidney disease, lymphocyte, neutrophil

## Abstract

We evaluated whether the neutrophil-to-lymphocyte ratio (NLR) could aid dialysis decision-making in combination with the clinical presentation and biochemical findings. We retrospectively evaluated the medical records of 279 patients who commenced chronic maintenance hemodialysis. We compared the laboratory findings at 6 months before dialysis to those at dialysis initiation. NLR cutoffs and risk factors for each of six uremic symptoms were determined. Mean age was 60.7 years and mean estimated glomerular filtration rate (eGFR) was 5.7 ± 2.5 mL/min/1.73 m^2^ at the time of hemodialysis and 7.7 ± 3.8 mL/min/1.73 m^2^ 6 months earlier (*p* < 0.001). The mean NLR increased significantly from 2.5 ± 1.0 to 4.9 ± 2.8 (*p* < 0.001). The NLR was positively correlated with the C-reactive protein level (*r* = 0.202, *p* = 0.009) and negatively correlated with those of albumin (*r* = −0.192, *p* = 0.001) and total CO_2_ (*r* = −0.134, *p* = 0.023). The NLR cutoffs for neurological and gastrointestinal symptoms as determined using receiver operator curve analysis were 2.4 (area under the curve [AUC] 0.976; 95% confidence interval [CI] 0.960–0.993; sensitivity 92.2%; specificity 94.7%) and 3.6 (AUC 0.671; 95% CI 0.588–0.755; sensitivity 68.1%; specificity 63.5%), respectively. On multiple linear regression analysis of neurological symptoms, the NLR was a significant predictor (*β* = −0.218, *p* = 0.017), as was age (*β* = 0.314, *p* = 0.037). In conclusion, the NLR may serve as a supplementary marker predicting uremic symptoms and a need for hemodialysis in stage 5 CKD patients.

## Introduction

The optimal timing of dialysis commencement in patients with stage 5 chronic kidney disease (CKD) has yet to be determined [1]. Having an optimal timing greatly improves prognosis. Excessively late initiation of dialysis is associated with high risks of mortality and hospitalization during the transition period and the next year [1–3]. The use of the estimated glomerular filtration rate (eGFR) to decide when dialysis should be initiated remains controversial, although this measure is well-established, convenient, and widely used [4–7]. In practice, dialysis initiation in clinical settings is based not on the eGFR but rather, careful observation of uremic symptoms and signs [8–10]. Markers other than the eGFR are needed to optimally time dialysis initiation; this reduces risks associated with delayed dialysis, the need for medical management, and mortality [1].

It is well-known that cardiovascular events, infections, anemia, and malnutrition commonly cause morbidity and mortality in patients with end-stage kidney disease (ESKD) [11–13]. The serum levels of inflammatory mediators such as C-reactive protein (CRP), tumor necrosis factor-alpha (TNF-*a*), and interleukin-6 (IL-6) are increased in CKD and dialysis patients [14–17]. The neutrophil-to-lymphocyte ratio (NLR) is an accepted surrogate marker of endothelial dysfunction and inflammation—thus, both micro- and macro-vascular complications—and is also significantly prognostic in CKD and dialysis patients [18–21]. An elevated NLR may be associated with poor outcomes of patients undergoing maintenance hemodialysis [20,22] and is a predictor of all-cause mortality and cardiovascular events in patients with CKD [23].

We frequently encounter significant NLR changes in patients commencing hemodialysis, regardless of residual renal function. We thus suspected that the NLR would reflect the development of uremic symptoms in stage 5 CKD patients. Hence, we explored whether changes in the NLR of such patients would help with predicting an optimal time for the initiation of dialysis. Furthermore, we determined whether disease severity as indicated by the NLR is associated with six categories of uremic symptoms. We suggest that the NLR may serve as a useful marker in stage 5 CKD patients who may need to commence dialysis.

## Methods

### Study population

**Figure 1.**
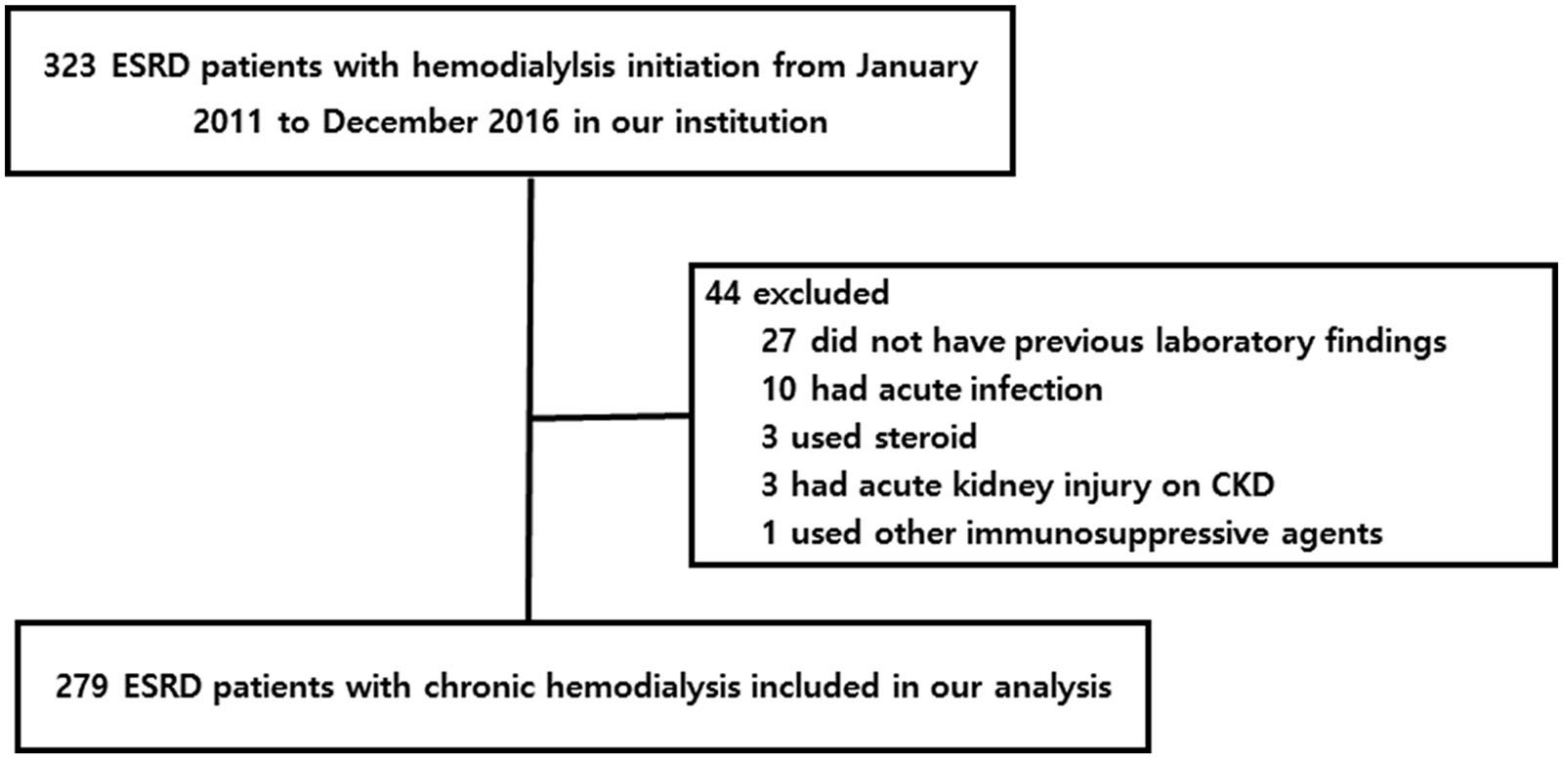
Overall workflow of patient’s enrollment.

The Institutional Review Board (IRB) of Gyeongsang National University Hospital (GNUH) approved this study (IRB no. IRB no. 2021-04-011) and waived the need for informed consent due to its retrospective study design. We retrospectively evaluated the medical records of patients who commenced chronic maintenance hemodialysis from January 2011 to December 2016. Patients aged 18–75 years were initially enrolled. Data on demographic and clinical characteristics, laboratory findings, and comorbidities were obtained from medical records. Patients with acute infections, on steroids or immunosuppressive agents, or on hemodialysis to treat acute kidney injuries were excluded. Patients for whom laboratory data at 6 months prior to dialysis initiation were lacking were excluded. A total of 323 ESKD patients on chronic hemodialysis were initially registered, and 44 were then excluded (Figure 1). We compared laboratory data obtained at 6 months before dialysis initiation to those at the time of initiation.

### The six categories of uremic symptoms

The symptoms of patients who commenced maintenance hemodialysis were divided into six categories. Neurological symptoms included mental changes, fatigue, headache, insomnia, and generalized weakness; pulmonary symptoms included dyspnea caused by uremic pleuritis and pulmonary edema caused by heart failure; gastrointestinal (GI) symptoms included anorexia, nausea, and vomiting; and dermatological symptoms included dry skin and pruritus. The last two categories were weight loss > 10% and intractable peripheral edema. Maintenance hemodialysis was applied to patients with CKD after evaluating above six uremic symptoms and signs, regardless of the eGFR

### Definitions

Automated complete blood counts (CBCs) and differential counts, total white blood cells (WBCs), neutrophils, and lymphocytes] were obtained, and the NLR was calculated as the ratio of neutrophils to lymphocytes. The eGFR was calculated using the Modification of Diet in Renal Disease formula [1.86 × (plasma creatinine) − 1.154 × (age) − 0.203)] × (0.74 if female) × (1.210 if black).

### Laboratory investigations

Venous blood was drawn from all subjects, and the levels of albumin, alkaline phosphatase (ALP), uric acid, total cholesterol, CRP, blood urea nitrogen (BUN), creatinine, calcium, phosphorus, electrolytes, and total CO_2_ were measured using an autoanalyzer.

### Statistical analyses

All data are presented as the means ± standard deviations or frequencies. The paired t-test was used to compare continuous variables recorded at 6 months before and at the time of chronic maintenance hemodialysis initiation. Dichotomous variables were compared using the chi-squared test. The relationships between the presence of symptoms in the aforementioned six categories and disease severity as indicated by the NLR were examined using the *t*-test. Linear associations between the NLR and other continuous variables were assessed using the Pearson correlation test. NLR cutoffs for the six categories of uremic symptoms were derived by drawing receiver operating characteristic (ROC) curves. Multiple logistic regression analysis was performed to identify independent risk factors for all six categories of uremic symptoms present at the initiation of hemodialysis. All statistical analyses were performed using SPSS for Windows (ver. 21.0; SPSS Inc., Chicago, IL, USA). Statistical significance was defined as *p* < 0.05.

## Results

### Clinical and biochemical parameters

**Figure 2.**
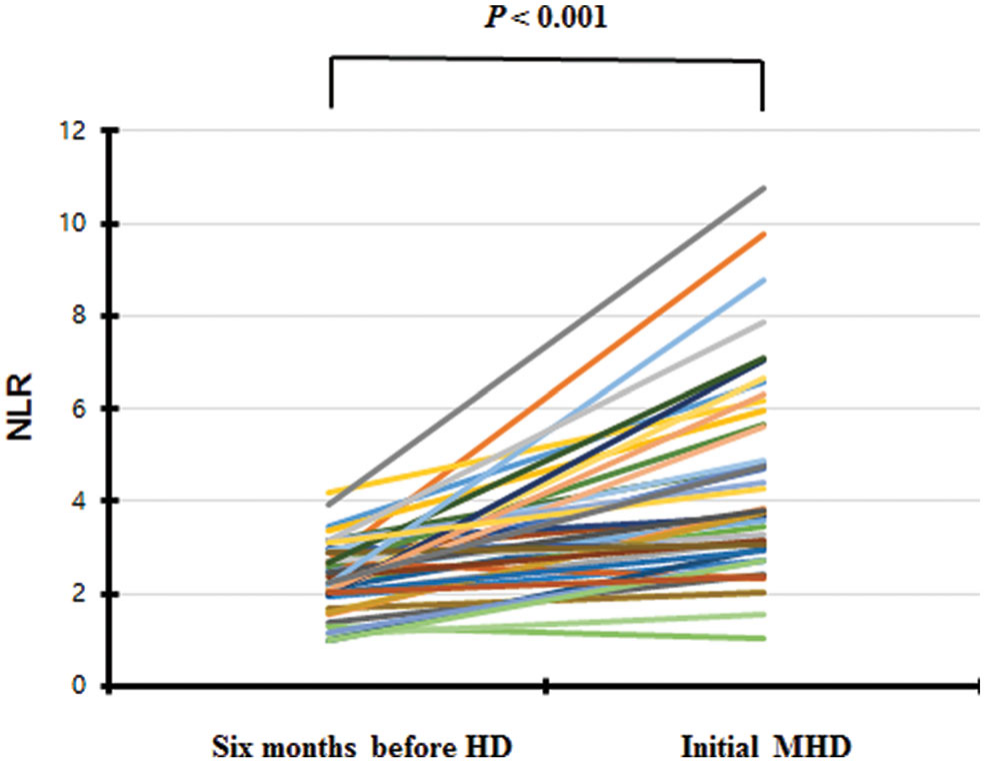
The change of NLR six months before and at HD initiation.

The clinical and biochemical characteristics of 279 ESKD patients at 6 months before and at dialysis initiation are summarized in Table 1. The mean age at admission was 60.7 years, and that at 6 months before dialysis initiation was 60.5 years; 68.5% of the patients were male. The ESKD etiologies were diabetic nephropathy (58.8%), chronic glomerulonephritis (26.5%), hypertensive nephropathy (11.1%), polycystic kidney disease (2.2%), and unknown (1.4%). The mean eGFR at dialysis initiation was 5.7 mL/min/1.73 m^2^ but 7.7 mL/min/1.73 m^2^ at 6 months prior (*p* < 0.001). The level of CRP (an acute-phase reactant) did not change over the 6 months, but the total WBC count increased significantly (*p* < 0.001). The hemoglobin, albumin, and total CO_2_ levels decreased significantly (all *p* < 0.001) (Table 1). The NLR increased from 2.5 ± 1.0 to 4.9 ± 2.8 (*p* < 0.001) (Figure 2). The NLR rose more than 50% in 70.1% of the patients.

**Table 1.** Clinical and laboratory characteristics before six months and at hemodialysis initiation.

Variables	Total (*N* = 279)	6 mo before dialysis (*N* = 279)	At MHD initiation (*N* = 279)	*p* value
Age (years)		60.5 ± 13.5	60.7 ± 13.4	0.324
Men (*N*, %)	191 (68.5)			
Primary disease				
Diabetic nephropathy, *n* (%)	164 (58.8)			
Hypertensive nephropathy, *n* (%)	31 (11.1)			
Chronic glomerulonephritis, *n* (%)	74 (26.5)			
Polycystic kidney disease, *n* (%)	6 (2.2)			
Unknown, *n* (%)	4 (1.4)			
Hemoglobin (12.0–16.0, g/dL)		9.1 ± 1.6	8.4 ± 1.5	<0.001
WBC (4.0–10.0, ×10^3^ /*u*L)		6.3 ± 2.0	7.4 ± 2.0	<0.001
Platelet (130–400, ×10^3^ /mm^3^)		205.0 ± 74.5	203.4 ± 84.7	0.673
Albumin (3.5–5.2, g/dL)		3.5 ± 0.7	3.3 ± 0.7	<0.001
Cholesterol (120–200, mg/dL)		160.2 ± 51.0	146.7 ± 48.1	<0.001
Uric acid (2.6–6.0, mg/dL)		8.4 ± 3.9	8.62 ± 3.6	0.169
BUN (8.0–20.0, mg/dL)		73.0 ± 21.4	91.0 ± 28.3	<0.001
Calcium (8.8–10.6, mg/dL)		8.3 ± 0.8	8.0 ± 1.0	<0.001
Phosphorus (2.5–4.5, mg/dL)		5.2 ± 1.4	5.9 ± 1.9	<0.001
ALP (30–120, U/L)		89.0 ± 52.1	87.0 ± 52.6	0.306
Sodium (135–145, mmol/L)		138.7 ± 3.6	136.3 ± 4.9	<0.001
Potassium (3.5–5.0, mmol/L)		5.1 ± 0.8	4.8 ± 0.9	<0.001
Total CO_2_ (21–31, mmol/L)		19.4 ± 8.2	16.5 ± 4.9	<0.001
CRP (0.0–5.0, mg/L)		7.5 ± 22.0	10.4 ± 26.6	0.296
Creatinine (0.5–1.0, mg/dL)		6.8 ± 2.6	8.9 ± 3.7	<0.001
eGFR (ml/min/1.73 m^2^)		7.7 ± 3.8	5.7 ± 2.5	<0.001
NLR		2.5 ± 1.0	4.9 ± 2.8	<0.001

WBC: white blood cell; BUN: blood urea nitrogen; ALP: alkaline phosphatase; CRP: C-reactive protein; eGFR: estimated glomerular filtration rate; NLR: neutrophil-lymphocyte ratio.

### Correlation analysis

**Figure 3.**
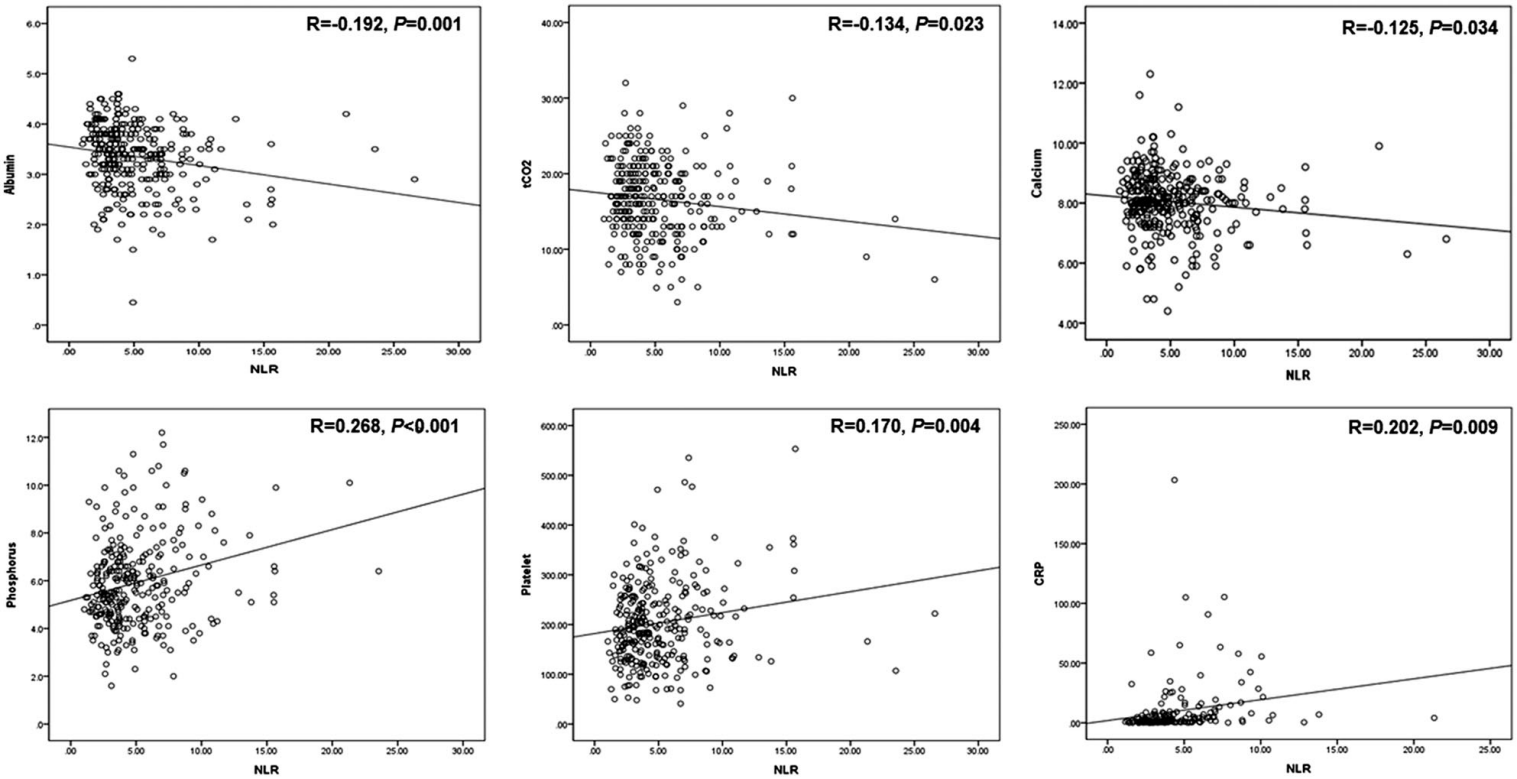
Scatter plot figures for positive and negative correlations of NLR.

On bivariate correlation analysis, the NLR was positively correlated with the CRP (*r* = 0.202, *p* = 0.009), platelet (*r* = 0.170, *p* = 0.004), and phosphorus (*r* = 0.202, *p* = 0.009) levels and inversely correlated with the albumin (*r* = −0.192, *p* = 0.001), calcium (*r* = −0.125, *p* = 0.034), and total CO_2_ (*r* = −0.134, *p* = 0.023) levels (Figure 3). We found no relationship between the NLR and eGFR (*r* = −0.058, *p* = 0.328), total cholesterol level (*r* = 0.058, *p* = 0.356), or hemoglobin level (*r* = −0.076, *p* = 0.199).

### Relationships between the NLR and the six categories of uremic symptoms

The mean number of uremic symptom categories for which symptoms were observed was 3.0 ± 0.9. Of the six categories, the percentage of patients presenting with symptoms from only one category was 2.8%; no patient complained of symptoms from all six categories. The percentages complaining of symptoms from 2, 3, 4, and 5 categories were 30.7, 35.9, 23.3, and 7.3%, respectively. Of all patients, 93.4% complained of neurological symptoms, 78.8% of GI symptoms, 44.6% of pulmonary symptoms, and 34.1% of dermatological symptoms. Weight loss and peripheral edema were evident in 5.2 and 39.7% of the patients, respectively. The NLR was significantly correlated with the mean number of symptom categories observed (*p* < 0.001).

#### NLR cutoffs for two categories of uremic symptoms

**Figure 4.**
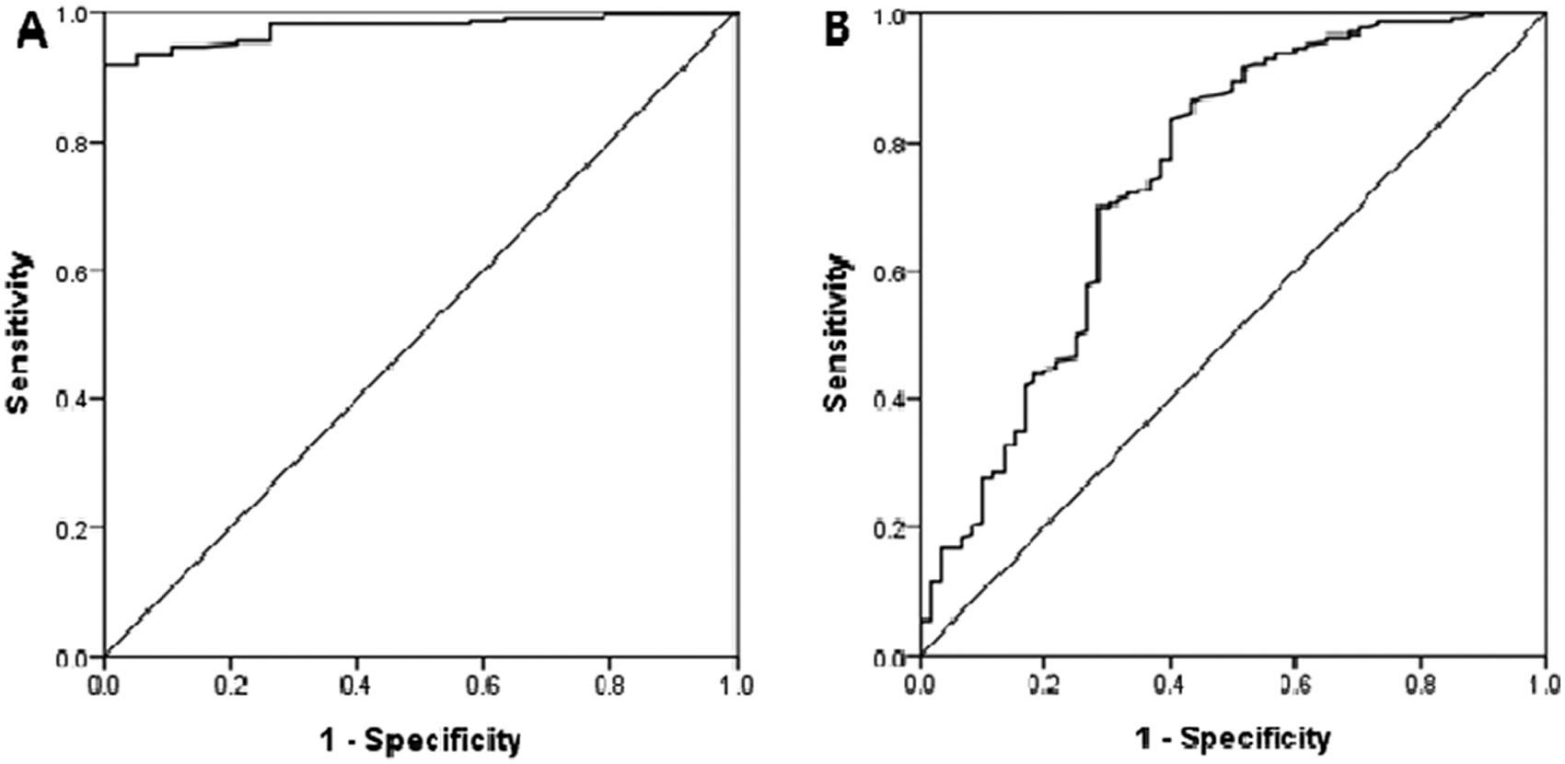
Receiver operator characteristic (ROC) curve between NLR and neurologic symptoms (A) and GI symptoms (B) to present at hemodialysis initiation.

The NLR cutoffs for neurological and GI symptoms (as determined using ROC curve analysis) were 2.4 [area under the curve (AUC) 0.975; 95% confidence interval (CI) 0.960–0.993; sensitivity 92.2%; specificity 94.7%] and 3.0 (AUC 0.747; 95% CI 0.669–0.825; sensitivity 84.1%; specificity 60.5%), respectively (Figure 4). It was impossible to determine cutoffs for the other four symptom categories because all AUCs were less than 0.5.

### Predictors of categories of uremic symptoms

Multiple logistic regression analysis of neurological and GI symptoms showed that the NLR was significantly predictive [*p* < 0.001, odds ratio (OR) 32.030, CI 6.412–160.0; *p* < 0.001; OR 1.583, CI 1.298–1.930, respectively] of whether these types of symptoms were present (Tables 2 and 3). However, multiple logistic regression analysis did not identify any factor that significantly predicted the presence of the other four categories of uremic symptoms (data not shown).

**Table 2.** Dependent risk factors to predict neurologic uremic symptoms.

	*p* value	*OR	95% confidence interval [CI] for OR	
			Lower	Upper
Age (years)	0.146	0.956	0.900	1.016
eGFR (ml/min/1.73 m^2^)	0.210	0.785	0.537	1.146
BUN (mg/dL)	0.922	0.998	0.968	1.030
tCO_2_ (mmol/L)	0.397	1.076	0.909	1.274
NLR	<0.001	32.030	6.412	160.0

eGFR: estimated glomerular filtration rate; BUN: blood urea nitrogen; NLR: neutrophil-lymphocyte-ratio; tCO_2_: total tCO_2_.

**Table 3.** Dependent risk factors to predict GI symptoms.

	*p* value	*OR	95% confidence interval [CI] for OR	
			Lower	Upper
Age (years)	0.696	0.995	0.973	1.019
eGFR (ml/min/1.73 m^2^)	0.592	1.043	0.894	1.217
BUN (mg/dL)	0.066	0.988	0.975	1.001
tCO_2_ (mmol/L)	0.885	0.995	0.930	1.065
NLR	<0.001	1.583	1.298	1.930

eGFR: estimated glomerular filtration rate; BUN: blood urea nitrogen; NLR: neutrophil-lymphocyte-ratio; tCO_2_: total tCO_2._

## Discussion

We found that the NLR significantly increased at the start of dialysis compared to six months before the dialysis initiation. The NLR was significantly correlated with CRP, serum albumin, and bicarbonate levels but not the eGFR. The NLR was also positively associated with the mean total number of uremic symptom categories observed. The NLR independently predicted the risk of neurological uremic symptoms and signs in stage 5 CKD patients. This is the first study on incident hemodialysis patients to demonstrate the complementary role of the NLR in terms of determining when to commence dialysis.

Nephrologists have reported high-level mortality and morbidity in the first 3 months after the initiation of dialysis [2,3]. Risk factors and appropriate interventions have been sought [1,8,9]. It has been speculated that dialysis timing affects mortality and morbidity. Only one randomized control study (but several large observational studies) has explored whether dialysis initiation based on the eGFR affected the subsequent clinical outcomes. The Initiating Dialysis Early and Late (IDEAL) study found that early dialysis initiation based on the eGFR did not enhance survival or clinically important secondary outcomes. However, the work had certain limitations [24]. The IDEAL study challenged the use of the eGFR as the principal guide for dialysis initiation and affected clinical practice in several countries [24,25]. We also emphasize the compelling need for measures other than the eGFR when seeking to optimally time the initiation of chronic maintenance dialysis, to (potentially) improve survival and clinical outcomes.

The Kidney Disease Improving Global Outcomes (KDIGO) 2012 guidelines on CKD evaluation and management state that decisions regarding the timing of dialysis initiation should rely primarily on the assessment of symptoms or signs attributable to kidney disease [26]. The guidelines also state that symptoms are to be expected at a GFR of 5–10 mL/min/1.73 m^2^ but do not recommend that this GFR should trigger dialysis regardless of the extent of uremic symptoms. The 2015 Kidney Disease Outcomes Quality Initiative (KDOQI) guidelines recommend that dialysis initiation should be based on the assessment of specific complications of CKD [27] but do not mention a specific GFR range. We usually commence maintenance dialysis after evaluating the uremic symptoms and signs of chronic renal failure, regardless of the eGFR. The eGFR on hemodialysis commencement in the present study was 5.7 mL/min/1.73 m^2^. The 2018 USRDS annual report indicated that this was the average eGFR among incident ESRD patients aged 18 years and older [28].

It is difficult to optimally time dialysis initiation based on the symptoms and signs of chronic renal failure because such symptoms are subtle, CKD patients usually adapt to a slow and progressive symptom burden, and such patients often do not report all symptoms [1]. No consensus method is available by which to accurately assess the grade/severity of uremic symptoms and signs in CKD patients under consideration for maintenance dialysis [9,29,30]. We usually assess the physical symptoms and signs of advanced renal disease, but emotional and psychological symptoms are also important when deciding whether to commence renal replacement therapy [31]. Few studies have explored the nature, severity, or prevalence of symptoms among patients with stage 5 CKD commencing maintenance dialysis [32,33]. We thus defined six uremic symptom categories and estimated their prevalence when hemodialysis was initiated. Neurological and GI symptoms were the most common.

Several reports have shown that that CKD and ESKD are chronic inflammatory conditions in which the levels of several inflammatory markers increase [14,15,21]. The NLR is an inflammatory marker that correlates well with the levels of other inflammatory markers including CRP, IL-6, TNF-*a*, and serum albumin (negative acute-phase proteins) [17,18]. The NLR was reported to predict CKD progression and cardiovascular morbidity and mortality [19,20,22]. We found that the NLR was significantly higher at dialysis initiation than at 6 months prior as well as negatively correlated with the albumin level and positively correlated with the CRP level at both times, but not correlated with the eGFR. An unexpected finding was that the total CO_2_ level (reflecting the serum bicarbonate level) decreased significantly on dialysis initiation and was correlated with the NLR. We found no significant correlation between the total CO_2_ and CRP levels but a positive correlation between the total CO_2_ level and the eGFR (data not shown), indirectly suggesting that NLR may be better than the CRP level for assessing metabolic dysfunction and that changes in the NLR, eGFR, and total CO_2_ level should be closely observed in patients with stage 5 CKD under consideration for renal replacement therapy.

We explored whether the NLR usefully predicted a need for dialysis initiation. The greater the number of uremic symptoms, the higher the NLR. The NLR predicted the presence of symptoms in two uremic symptom categories; the AUCs for the other four categories were low. Multivariate analysis showed that the NLR significantly predicted the presence of neurological and GI symptoms. An NLR of 2.5 at 6 months before dialysis initiation was associated with the presence of uremic syndrome. However, these are difficult to assess in CKD patients because they are very subtle, CKD patients adapt to uremic burdens, and such patients (especially older patients) often do not complain of symptoms [34]. It is near-impossible for patients to elaborate on symptoms and signs and for physicians to accurately record them during short outpatient sessions. Therefore, it may be to be helpful to measure serial NLR changes and carefully assess the presence/absence of uremic syndrome when deciding when to commence dialysis. The NLR is readily available and inexpensive to measure.

Although hypocalcemia and hyperphosphatemia is usually late event in CKD due to adaptations, altered mineral metabolism defined as CKD-mineral bone disease (MBD) occurs much earlier [35]. Previous study showed that serum calcium and phosphate level was not significantly different in high and low NLR patients with stage 1–4 CKD [19]. In our study including stage 5 CKD patients, these levels have changed significantly with increase in NLR in the last 6 months. Furthermore, the NLR was positively correlated with phosphate level in our patients whereas inversely with calcium level. These correlations suggest that higher values of NLR to start maintenance hemodialysis might be associated with the severity of CKD-MBD. The clinical importance of correlation between NLR and CKD-MBD including serum calcium and phosphate level in ESRD patients to start maintenance hemodialysis remains to be determined.

Our work had certain limitations. First, this was an observational, retrospective, single-center study. All findings relied on medical records. We thus could not define cause-and-effect relationships. Second, we measured the NLR for each patient only twice; serial measurements would have been better, as they might have revealed changes in the 6 months prior to dialysis. Third, we limited the uremic symptoms evaluated on hemodialysis initiation to six categories. Fourth, it is always possible that hidden confounders were in play. Strengths of our study include continuous patient follow-up for least 6 months, identical laboratory procedures, and constant review of chronic management and dialysis indications.

In conclusion, the NLR is easily available and may serve as a useful auxiliary parameter predicting a need for dialysis in stage 5 CKD patients in combination with the eGFR; bicarbonate, CRP, and albumin levels; and uremic symptoms and signs. However, further research is needed, as are randomized controlled studies or well-designed cohort studies that identify, assess, and rate uremic symptoms.

## Data Availability

The datasets used and/or analyzed during this study are available from the corresponding author on reasonable request.
